# KASPar SNP genetic map of cassava for QTL discovery of productivity traits in moderate drought stress environment in Africa

**DOI:** 10.1038/s41598-021-90131-8

**Published:** 2021-05-28

**Authors:** Favour Ewa, Joseph N. A. Asiwe, Emmanuel Okogbenin, Alex C. Ogbonna, Chiedozie Egesi

**Affiliations:** 1 Department of Plant Production, Soil Science and Agricultural Engineering, School of Agriculture and Environmental Sciences, University of Limpopo, Sovenga, South Africa; 2 African Agricultural Technology Foundation (AATF), Nairobi, Kenya; 3 Department of Plant Breeding and Genetics Section, School of Integrative Plant Sciences, Cornell University, Ithaca, NY 14853 USA; 4 Cassava Breeding Unit, International Institute of Tropical Agriculture (IITA), Ibadan, Nigeria

**Keywords:** Biotechnology, Genetics

## Abstract

Cassava is an important staple in Sub-Sahara Africa. While its production has rapidly expanded to the dry savannahs of the continent, productivity is low in this ecology due to drought by farmers, extending the growth cycle from 12 months to 18, and sometimes 24 months to ensure better harvests. Yield is a complex trait and often difficult to manipulate for genetic gain in conventional breeding. Unfortunately, the dearth of molecular tools for decades has hampered molecular breeding (MB) to improve cassava productivity. This study was conducted to explore KASpar SNPs to generate more molecular tools to enhance genetic dissection of elite African germplasm for improved cassava productivity in dry environments of Africa where molecular resources are highly limited for crop improvement. To aid molecular genetic analysis of traits, a linkage map covering 1582.8 cM with an average resolution of 3.69 cM was constructed using 505 polymorphic SNP markers distributed over 21 linkage groups. Composite interval mapping using 267 F_1_ progeny in initial QTL mapping identified 27 QTLs for productivity traits in the dry savannah of Nigeria. The availability of KASPar SNPs are anticipated to improve the implementation of MB for the development of high performing drought-tolerant cassava varieties in Africa.

## Introduction

Cassava is an important food and starch crop, with excellent adaptability to multiple environments typically grown from 18 to 24 months in the dry environments. It is one of the most important source of energy in the diet of Africans^1,2^. Cassava has been historically grown in the humid and sub humid zones of Africa where amount of rainfall distribution is between 1000 and 2200 mm^3,4^. In recent years, cultivation has expanded to transitional belts/savannas with increasing need for more food (due to population pressures). The rapid expansion of cassava production into non-traditional ecologies of the savannas in the last two or more decades have necessitated need to breed for more adapted varieties. The most limiting abiotic constraint to cassava production is drought^2,5^. Development of varieties that can withstand moderate stress or sever stress conditions are critical to enhance higher productivity in marginal environments. Breeding for such complex traits can be very challenging due to its multigenic nature given that drought tolerance/productivity under stress is a complex phenomenon as it is driven by several morphological, physiological and yield-component traits^6,7^. Although, classical breeding has resulted in moderate improvement of cassava for several traits up to the late 90s, this often not at efficient pace leading to long breeding cycle. Advancement of biotechnology has resulted in the development of molecular tools to increase breeding efficiency in crops. Cassava benefited from such molecular tools leading to the development of the first molecular map^8^. This soon resulted in the development of several molecular markers such as RFLP, ESTs, RAPDs, ESTs and SSRs^8–18^. The integration of the early developed molecular markers for cassava has fast tracked the discovery of novel genes such as CMD1, CMD2, CMD3^18–21^ that has improved breeding and development of CMD resistant varieties. However the strong limitations attached to the earlier molecular markers were a drag in the effective dissection of traits in cassava with a large paucity of markers which did not permit good genome coverage and prediction of crop performance in the first set of genetic maps developed for cassava up to 2010 as SNPmarkers hadn’t been developed for this crop. The probability to find high polymorphisms around target genes has significantly increased with SNPs given that they are highly abundant in the genomes and provide highest map resolution when compared with other marking systems^22^. The development of SNPmarkers was considered very critical to improve a better understanding of cassava genetics and accelerate genetic improvement of cassava. The CGIAR Generation Challenge Programme (GCP) facilitated the development of KASP (Competitive allele Specific PCR) SNPs for cassava which resulted in access to 2000 KASPar SNPs to improve molecular breeding application via MASor MARs. This is also very crucial for Africa where the capacity and molecular resources to undertake development of molecular breeding are limited. This set of SNPs represented the first major and abundant size of SNPs available in the public domain for use by national and international programs. SNPs are rapidly revolutionizing genetic improvement of complex traits for prediction and selection accuracy in many crops. These markers are therefore critical to manipulate cassava improvement. In addition genetic mapping for productivity/drought tolerance in marginal lands in African germplasm have yet to receive intense studies in Africa. The objectives of this study were therefore to (1) develop KASP SNPs based genetic map of cassava using elite African parental non-inbred varieties adapted to moderate moisture stress; (2) identify key productivity/drought tolerance traits under moderate stress conditions of a 12 month growth cycle) (3) conduct QTL mapping to detect QTLs for adaptation, productivity and drought tolerance and then define pathways of exploring these QTLs to generate high performing cassava varieties in national programs of Africa.

## Materials and methods

### Development of mapping populations

Based on earlier existing data on germplasm evaluated, the mapping population was formed by crossing two parents TMS 98/0505 (female) and TMS98/0581 (male). The population (planting materials) used were developed by the authors of this study.TMS98/0505 (with 97 DTP REP2 as a pedigree) is an elite cassava genotype that was officially released in Nigeria in 2005. It has high dry matter and starch content, high CMD resistance, and high yielding with moderate flowering and early bulking abilities. The female parent TMS98/0581 whose pedigree is MPR POP REP 1 is a released cassava variety with high dry matter content, CMD resistance, yielding, stay green, drought tolerance and profuse flowering ability. Cassava has no inbred lines, so both parents are heterozygous. They have been selected for variation in morphological characters and other attributes such as stay green and adaptation (with TMS98/0505 being more adapted) in the dry savannas of Nigeria. The parents have also been selected for key target traits (good yield, high dry matter content and starch content) of breeding importance as these traits are intended to be co-selected with drought tolerance since the best F1 genotypes are planned for further use in breeding program. Given that the crop is highly heterozygous and shows multi-allelism, they are expected to segregate even for these key breeding target traits whose QTLs would be useful. The other trait of immense consideration was CMD for which both parents are resistant as both parents are believed to explore the CMD2 dominant gene as part of their genetic resistance to the disease. The materials used and the processes adopted with the studies are in full compliance with the institutional, national and international guidelines and legislation.

### Mapping population nursery

Seedlings were planted in jiffy pots in the screen house at Umudike. One month after planting they were transplanted to the field as field nursery to evaluate for diseases and generate enough stem cuttings to start field trial for QTL mapping. Umudike is a high disease pressure zone for cassava. It is located on the latitude 5°29′N, longitude 7°24′N, altitude 120 m, annual rainfall 2200 mm, annual temperature is 26 °C, relative humidity 50–95% and dystric luvisol as soil type. The seedling nursery was intensively managed at Umudike by weeding the field to ensure good establishment of the population which was critical to generate enough planting materials as cassava is clonally propagated. The vigorous genotypes in the nursery generated an average of 10 cuttings with some having much higher number of cuttings. This step was very important as it generated the cuttings which were used for the successful conduct of QTL mapping in Minjibir Kano State, Nigeria. Minjibir is a low disease pressure zone for cassava and wasn’t deemed suitable to assess the genetic response of the mapping population to diseases. The disease evaluation data from Umudike was to complement other agronomic traits evaluated in Minjibir under moderate stress conditions.

### Description of field experimental site

The genotypes were evaluated in Minjibir, Kano (sub-optimal soil conditions). Kano is in the Sudan savannah ecology with 2–3 months of rainfall in a year. This makes it suitable for drought studies. It is located at latitude 12°3′N and Longitude 8°32′E. The altitude is 473 m above sea level. Temperature at the location ranges from 18.7 to 66.5 °C while annual temperature is 41.9 °C. Relative humidity ranges from 13 to 68% while annual relative humidity is 31.1%. Rainfall range is from 0 to 320 mm while average annual rainfall is 270 mm which is sub-optimal for good plant growth with high risk (25–75%) of crop failure.

### Experimental design and trial

The experiment was for a moderate stress conditions for at least 6 months of stress (period without rainfall). The F1 progeny of 267 genotypes was evaluated for drought tolerance and productivity traits in Minjibir, Kano. The parents (TMS98/0505 and TMS98/0581) and three other elite cassava genotypes for different adaptation levels for dry ecology were selected (TMS91/02324 with low adaptation, TMS30572 with moderate adaption and TME419 with high adaptation) were used as check. Cassava cuttings of uniform length (20–30 cm) were planted in an augmented design. Augmented design was used due to limited supply of planting materials to permit replication. A single row planting of five plant stands per plot was done. The stakes were horizontally placed in the soil at the recommended 1 m spacing within plants per row and 1 m separation between the rows. The trial was established in the dry season and supported with supplementary irrigation for the first 3 months to enhance homogenous germination, sprouting and plant establishment and was then subjected to natural weather conditions (with 2 months of rainfall) in the ecology during a 12-month growth cycle.

### Phenotyping

The mapping population (Population B) was established for phenotyping studies for key traits linked to drought tolerance and high productivity in the dry ecologies. For phenotyping activities, data for several traits were collected in the field. There are several traits thought to be associated with drought tolerance in cassava^23,24^, although it is not known to what extent these traits vary in different genetic background in driving adaptation and drought tolerance. The following traits were evaluated based on standard procedures used for phenotyping in cassava:

- Morphological characters—These include: plant height; first branching height (HFB), height of leafless stem, plant vigour; branching level; number of leaf scars, leaf retention and, number of leaves
- Productivity traits (root yield and related characters)—These include root weight, biomass, root number, fresh root yield, harvest index, dry matter content and dry root yield,. They were measured during harvest at 12MAP. The estimation of percentage dry matter content (DMC) was determined using the specific gravity method described by^25^. Approximately 1 to 3 kg of roots were weighed in air (W_A_) using a hanging scale and then the same sample was weighed in water (W_W_).
  $$specific \; gravity=\frac{weight \; in \; air}{weight \; in \; air-weight \; in \;water}$$

Dry matter content was estimated using the formula:

$$\% {\text{ DMC }} = \, \left( {{158}.{3}} \right) \, *\left( {{\text{W}}_{{\text{A}}} /{\text{W}}_{{\text{A}}} - {\text{W}}_{{\text{W}}} } \right) \, - {142}.0) \, *{1}00$$

Dry root yield (DRY) was derived by multiplying FRY with percentage DMC.

Cassava mosaic severity was assessed during the crop growth at 3, 6 and 9 months after planting on a scale of 1–5 where severity increases along the scale.

### Genotyping

KASPar SNP markers developed for cassava in another GCP linked project at the University of Maryland and IITA were validated and subsequently converted to KASPar based platform. KASPar is flexible and is based on competitive allele-specific PCR. KASPar is designed to query one SNP at a time. A total of 2000 SNPs were made available and 94% (1845) were successfully converted to LGC system. DNA was extracted from freshly harvested leaves for 256 genotypes of the population B and parents using LGC extraction kits. The parents were surveyed for polymorphic SNP markers and the informative markers were then used to genotype mapping populations at the LGC Genomics Laboratory. The KASP genotyping assays were based on competitive allele-specific PCR and were used to bi-allelically score the SNPs at specific loci. The KASP Assay mix contains three assay-specific non-labelled oligos: two allele-specific forward primers and one common reverse primer. The allele-specific primers each harbour a unique tail sequence that corresponds with a universal FRET (fluorescence resonant energy transfer) cassette; one labelled with FAM dye and the other with HEX dye. The KASP-TF Master Mix contains the universal FRET cassettes, ROX passive reference dye, taq polymerase, free nucleotides and MgCl_2_ in an optimized buffer solution. During thermal cycling, the relevant allele-specific primer binds to the template and elongates, thus attaching the tail sequence to the newly synthesized strand. The complement of the allele-specific tail sequence is then generated during subsequent rounds of PCR, enabling the FRET cassette to bind to the DNA. The FRET cassette is no longer quenched and emits fluorescence. Competitive allele-specific PCR achieves bi-allelic discrimination through the competitive binding of the two allele-specific forward primers. If the genotype at a given SNP is homozygous, only one of the two possible fluorescent signals will be generated. If the genotype is heterozygous, a mixed fluorescent signal will be generated.

Complete details on principle and procedure of the assay on LGC genotyping high throughput platforms can be assessed using the link (http://www.lgcgenomics.com). FlapJack software was used for interactive visualization of high-throughput genotype data to check the data quality, marker information across loci for each F1 genotype and across population per marker. Segregation of the KASPar SNP markers were viewed graphically using SNP viewer. The markers were tested for goodness of fit to the expected Mendelian ratio following Chi square test (i.e. test for deviation from expected Mendelian segregation for each marker) and the best segregating and informative markers were used in mapping. Genetic linkage map was calculated with SNP markers using “CP option” of JoinMap Version 4.1^26^. JoinMap 4.1 was used to find the order of the markers in the linkage groups. Following the calculation of pairwise recombination frequencies, linkage groups were identified using LOD values ranging from 3 to 7 and ‘Kosambi’ mapping function was used to calculate centimorgan (cM) distances.

### Statistical analysis

The field data collected was subjected to analysis using Microsoft Excel and SAS software (version 9.0). Pearson’s phenotypic correlation analysis was used to determine traits that are significantly associated and which may aid better understanding of plant performance for drought tolerance and adaptation. Principal component analysis was used to determine the traits that were main contributors to drought tolerance and productivity traits. Simple statistics using Excel application was used to assess standard deviation, mean of the traits across the genotypes and coefficient of variation. Frequency distribution was used to assess variation among the traits and to determine the transgressives in the population.

### Marker-trait analysis and QTL mapping

QTL analysis was initiated with the phenotypic data of Population B starting with single marker regression analysis using R/QTL based on a LOD threshold of ≥ 3.0. Interval mapping (IM) and composite interval mapping with Bayesian model was used for QTL detection through R/qtl V1.37-11. Haley-Knott regression approach in R/QTL was also explored for multiple QTL analysis. The peak, map position, confidence interval, estimated effects of the QTLs, phenotypic variation (PVE), additive (A) and dominance components for each QTL were determined during the analysis. Digenic interactions were also determined using composite interval mapping analysis. The D/A ratio of 0–0.20, 0.21–0.80, 0.81–1.20, or > 1.20 was used to determine additive (A), partial dominance (PD), dominance (D) and over dominance (OD) mode of gene action respectively as described by^27^. Analysis of the QTLs was carried out using R/qtl software package^28^.

## Results

### Development of the mapping population

The cross between TMS98/0505 and TMS98/0581 (Population B parental lines) gave good flowering to generate seeds. There was good genetic variation in plant formation at the nursery. While a high number of the seedlings showed good development, many of the genotypes had extremely stunted growth. Once a genotype could support some cutting development (irrespective of its vigor) and they were included as part of the population to support wide genetic variation required for good QTL mapping. The extremely weak genotypes in the nursery which couldn’t generate sufficient cuttings to provide adequate number of plants per genotype and replications for field trials necessary for QTL mapping were unavoidably dropped. Results indicate that over 200 genotypes in the population showed plant development that could have at least 8–10 cuttings irrespective of its vigor and constituted the mapping population used for this study. These genotypes had very good variation in plant development and vigor to support mapping for positive and negative alleles.

### Phenotypic data analysis

#### Major limiting disease to plant performance: Cassava Mosaic Disease (CMD)

The mapping population which was evaluated for CMD disease in Umudike at specific intervals (3, 6, 9 MAP) showed that the distribution for CMD resistance was very good for most of the genotypes (above 90%) in the population. The parental lines used for the mapping population were resistant as typically characterized for the genotypes. A major proportion of the population either showed high resistance or resistance to the disease. Only a few genotypes (less than 5%) were susceptible to CMD, and they were dropped to avoid confounding effects of the disease on plant performance and QTL mapping.

#### Quantitative variation

**Figure 1:**
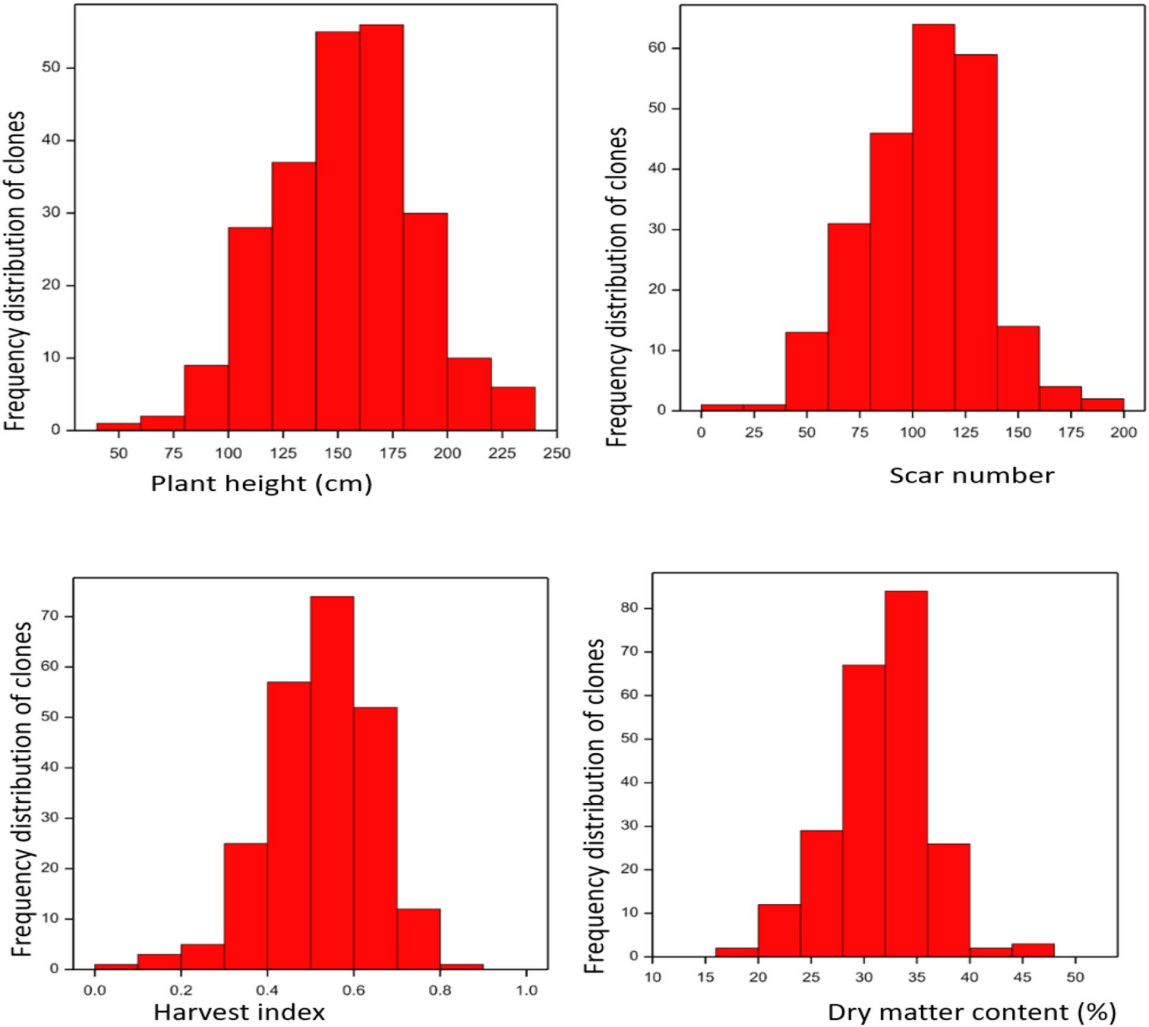
Frequency distribution of productivity and morphological traits.

The result of the phenotypic statistics, including mean, skewness, minimum and maximum are shown in supplementary data. The data suggest that there was good phenotypic variation for the physiological, morphological and productivity traits evaluated in the population (Table 5 supplementary data) All ranges of vigor were found in the population (scores from 1 to 5). There was also high variation in plant development as captured for example by plant height a range showing a height difference of over 200 cm among some genotypes. With respect to productivity traits such as dry matter content there was also big range (with 24% difference between the lowest and the highest values). Similarly, for dry root yield, differences were as high as 26t/ha. Results indicate good genetic variation across traits to support QTL mapping. Frequency distribution indicates that some of the traits segregated continuously (Fig. 1) and skewness values suggested that some of the traits in the present study were normally distributed and thus suitable for QTL mapping. The standard deviation observed indicates good degree of dispersion of the measurements for most of the traits. It was also observed that mean values of progenies for most of the traits were higher than that of the parents except for number of leaves, harvest index and dry matter content (Table 6 supplementary data).

#### Trait correlations

Pearson correlation coefficient analysis is shown in Table 1. Fresh root yield (FRY), which reflects economic productivity was positively correlated with many traits. It was highly and significantly correlated with dry root yield < 0.0001; r = 0.96). FRY was also moderately and significantly correlated with harvest index (r = 0.46). Harvest index correlated positively and significantly with key productivity traits such as dry matter content. Dry matter content which is a measure of the active utilizable component of productivity was positively correlated with dry root yield, plant height, length of leafless stem, plant vigour, scar number, branching level and root number and all were significant. Dry root yield which is the final target of production systems was positively and significantly correlated with key traits such as total plant biomass, plant vigour and root number that may signify breeding selection parameters. Other agronomic traits that were found to have correlated positively with dry root yield include leaf number, length of stem with leaves and branching level. For morphological expression, correlation analysis identified traits strongly connected. Plant height was strongly and significantly associated with length of leafless stem (r = 0.86), plant vigour (r = 0.69). Leaf number which is a measure of the photosynthate generation capacity was correlated positively with plant vigour, total plant biomass, root weight and root number. Scar level which could be a measure of stress avoidance mechanism (from leaf drop) had a positive correlation with most of the yield related traits. Root weight, total plant biomass, plant vigour and root number significantly and positively correlated with each other.

**Table 1:** Trait correlation of physiological and productivity traits in marginal environment.

Traits	FRY	HI	DMC	DYLD	PLTHT	SCARNO	LEVNO	SCARLEV	WTLV	WOTL	RTWT	BIOM	VIG	RTNO	CMD	CBB	BRLEV
FRY	1																
HI	0.46***	1															
DMC	0.03ns	0.25***	1														
DYLD	0.96***	0.51***	0.25***	1													
PLTHT	0.05ns	− 0.30***	0.15*	− .07ns	1												
SCARNO	0.16**	− 0.04ns	0.10ns	0.16ns	0.55***	1											
LEVNO	0.35**	0.07ns	− 0.01ns	0.31***	0.26***	0.26***	1										
SCARLEV	− 0.02ns	− 0.36***	0.15*	0.01ns	0.85***	0.58***	0.18**	1									
WTLV	0.30***	0.03ns	− 0.18**	0.22***	− 0.01ns	− 0.09ns	0.06ns	0.89***	1								
WOTL	− 0.04ns	− 0.28***	0.27***	0.01ns	0.86***	0.56***	0.19**	− 0.17**	− 0.24***	1							
RTWT	0.71***	0.15*	− 0.03ns	0.64***	0.24***	0.24***	0.21***	0.15*	0.30***	0.10ns	1						
BIOM	0.37***	− 0.36***	− 0.08ns	0.30***	0.48***	0.30***	0.22***	0.41***	0.24***	0.39***	0.74***	1					
VIG	0.18**	− 0.20**	0.20**	0.22***	0.69***	0.49***	0.31***	0.66***	− 0.02ns	0.28***	0.30***	0.54***	1				
RTNO	0.31***	0.03ns	0.12*	0.31***	0.35***	0.26***	0.20***	0.31***	0.06ns	0.73***	0.65***	0.64***	0.43***	1			
CMD	− 0.09ns	− 0.02ns	0.061ns	− 0.08ns	0.02ns	0.011ns	− 0.01ns	0.05ns	− 0.05ns	0.04ns	− 0.11ns	− 0.07ns	0.02ns	− 0.02ns	1		
CBB	− 0.22ns	− 0.17ns	− 0.08ns	− 0.22ns	− 0.14*	− 0.17**	− 0.27***	− 0.03ns	− 0.09ns	− 0.08ns	− 0.23***	− 0.17**	− 0.16**	− 0.18**	0.09ns	1	
BRLEV	0.20**	− 0.02ns	0.06ns	0.20**	0.22***	0.14*	0.33***	0.17**	0.03ns	0.20**	0.15*	0.37***	0.37***	0.15*	0.09ns	− 0.01 ns	1

FRY = fresh root weight (t/h), HI = harvest index, DMC = dry natter content (%), DYLD = dry root yield (%), SCARNO = scar number, SCARLEV = scar level, WTLV = length of stem with leaves (cm), WOTL = length of leafless stem (cm), RTWT = root weight (kg), BIOM = total plant biomass (kg), VIG = plant vigour, CMD = cassava mosaic disease, CBB = cassava bacterial blight, BRLEV = branching level, *P = ≤ 0.05, **P = ≤ 0.001, *** = P ≤ 0.0001.

#### Principal component analysis

Results based on five PCAs accounted for at least 74% of the variation for physiological traits and 99% variation for productivity traits. The first three PCs identified scar level (length of portion of stem without leaves, leaf retention, stem portions with leaf number of leaves and branching level as main contributors in the physiological traits while the first three PCs identified fresh root yield, dry root yield, root number, dry matter content and harvest index as main contributors for productivity traits.

### Genotyping and construction of genetic linkage map

**Figure 2:**
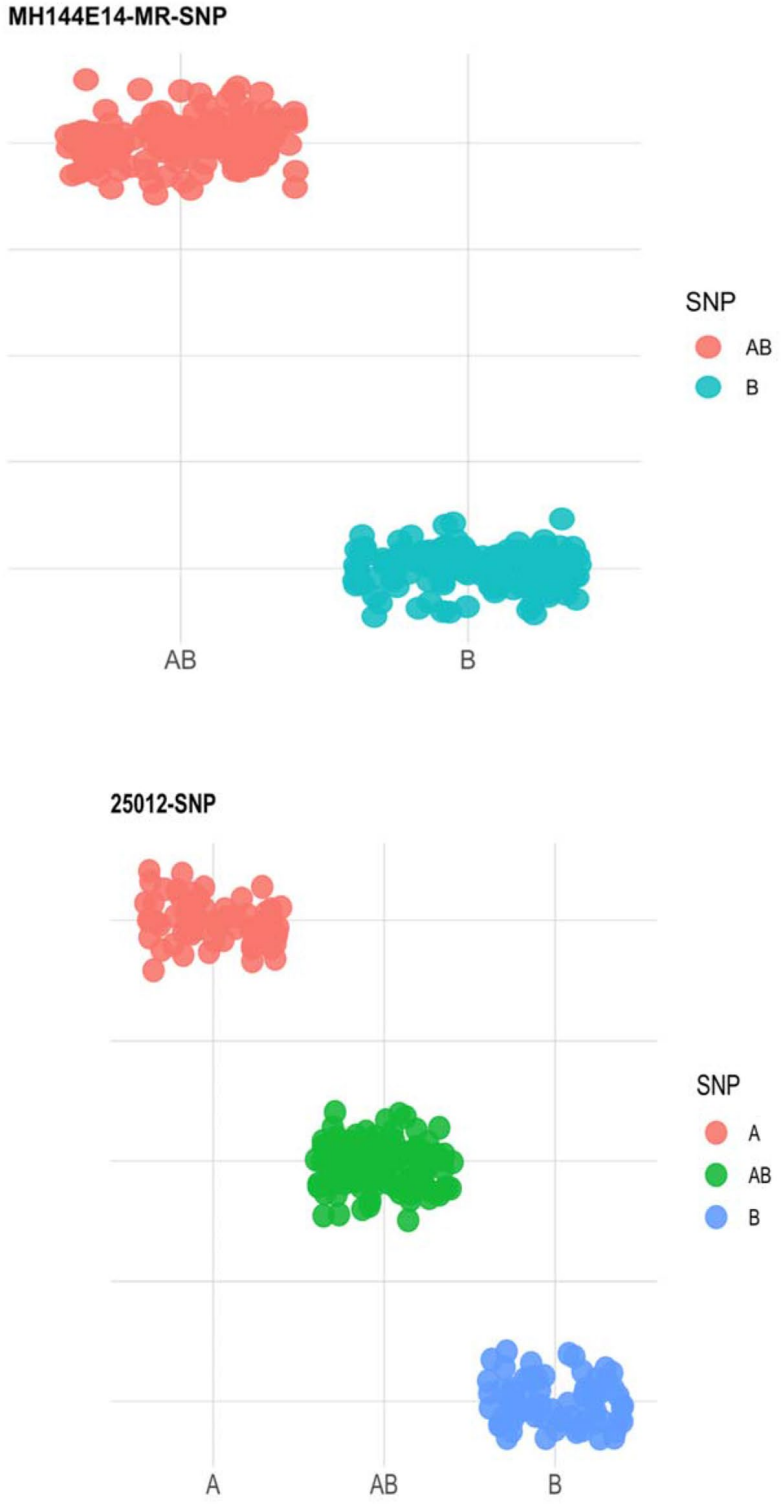
Marker segregation types in mapping population B.

**Figure 3:**
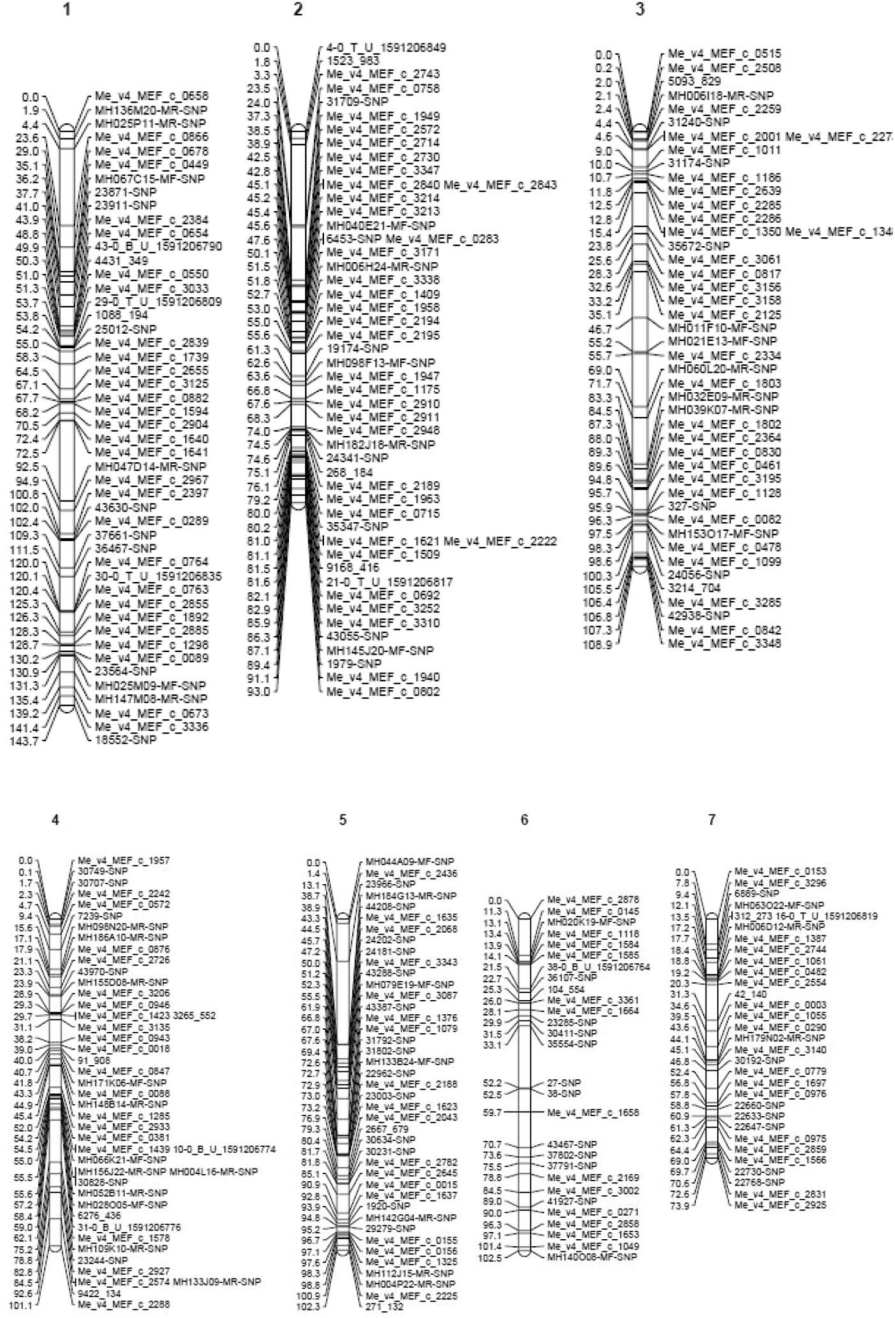

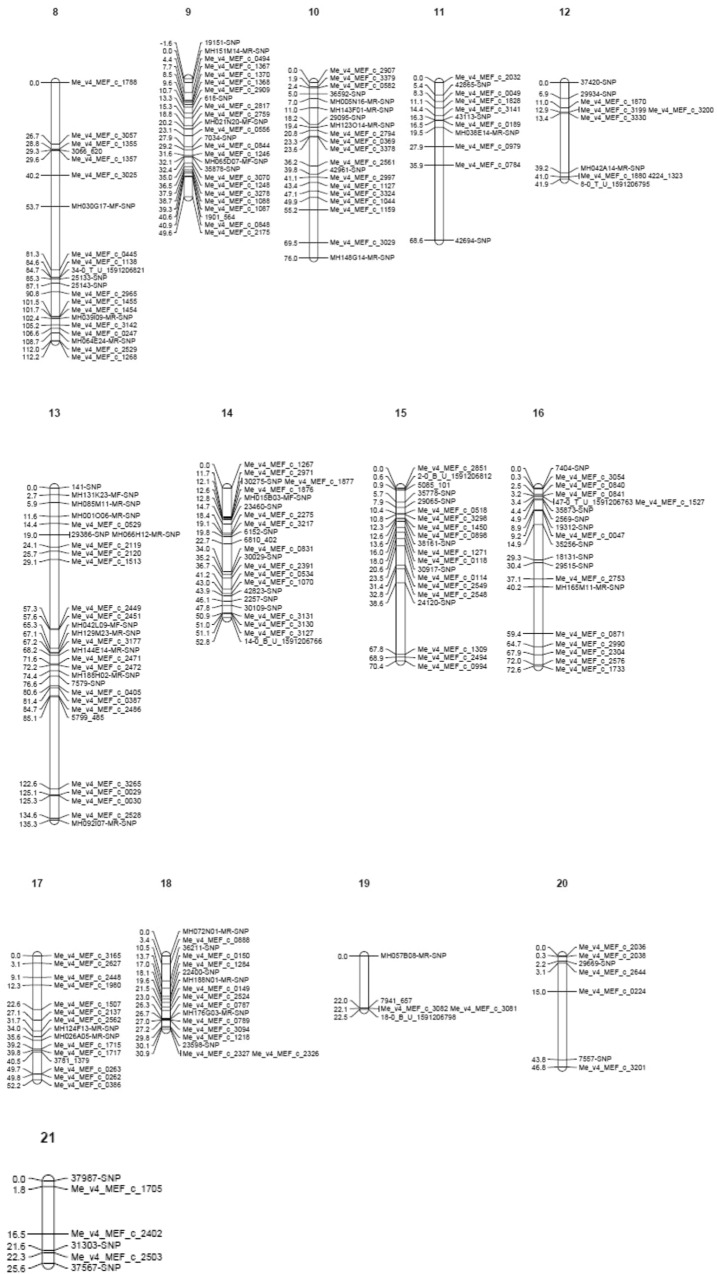
Genetic Linkage Map of cassava for mapping population B.

Genotyping of the mapping population with KASPar SNP array resulted in polymorphic markers showing either 1:1 or 3:1 segregation (Fig. 2). The polymorphic markers for the cross combination (parental pairs) selected for the development of mapping populations were also estimated to determine the total number of markers informative to be used for QTL mapping in family B mapping population. Results of parental survey with SNPs indicate that the number of polymorphic markers in each parent was between 522 and 576 markers while that of the cross was 856 markers. After removing ambiguous and unlinked markers, the genetic linkage map was constructed with 505 markers with fairly good distribution and coverage across all 21 linkage groups (synonymously used as chromosome in this paper) spanning 1582.8 cM in length with an average marker density of 3.69 cM (Fig. 3). Linkage group (LG) 1 was the longest (143.7 cM) while LG 20 was the smallest (22.5 cM). The number of markers per linkage groups ranged from 6 to 48 markers while the length of the linkage groups ranged from 25.6 to 143.7 cM with inter-marker distance ranging from 1.92 to 6.69 cM.

### QTL identification

Marker—trait linkage and composite interval analysis of population B resulted in the identification for evaluated traits associated as critical to adaptation for drought stress and productivity for the marginal environment targeted for this study. In all 27 QTLs were identified in eleven linkage groups (LGs 1, 2, 3, 4, 5, 6,7,10, 13, 18 and 21). Significant peak values of LOD scores (LOD ≥ 3), the position of these peaks, the percentage phenotypic variance explained, and the estimated gene actions are shown in Table 2. The number of QTLs identified per linkage group ranged from 1 to 5. The identified QTLs were found in 11 traits. Results indicate that dry root yield had the highest number of detected QTLs in this analysis.

**Table 2:** Mapping QTLs for physiology and morphological traits in cassava using composite interval mapping.

Trts^a^	QTL^b^	Chro^c^	Flanking markers	Position	LOD	Add^d^	Dom^e^	Gene action	%PVE^f^	P-value^g^
Plant height	*c18.loc19*	18	mk491-mk496	19.00	3.85	6.27	36.48	OD	7.296	0.0002***
Height at first branching	*c1.loc68*	1	mk005-mk026	68.0	3.06	1.72	− 19.50	A	4.495	0.002**
	*c3.loc15*	3	mk113-mk116	15.0	3.44	7.99	− 14.67	A	4.865	0.001**
	*c7.loc35*	7	mk272-mk274	35	4.77	83.49	− 77.66	A	7.802	2.88e−05***
Branching level	*c13.loc11.6*	13	mk381-mk383	11.6	25.85	− 0.10	− 20.08	OD	19.19	< 2e−16***
	*c21.loc0.0*	21	mk515-mk516	0.0	26.91	829.48	− 829.83	A	20.42	< 2e−16***
Dry matter cont	*c3.loc67*	3	mk122-mk126	67	3.89	1.9452	− 1.5500	A	7.67	0.0001***
Dry root yield	*c1.loc54*	1	mk015-mk035	54	3.01	1.62	1.00	PD	3.320	0.007678**
	*c3.loc68*	3	mk124-mk128	68	4.21	− 1.16	− 0.94	D	3.703	0.004440**
	*c4.loc57.2*	4	*mk175-mk181*	57.16	4.48	− 1.27	− 2.42	OD	5.940	0.0002***
	*c7.loc13*	7	*mk263-mk264*	13.00	4.15	− 1.59	7.37	A	4.390	0.002**
	*c10.loc2.4*	10	*mk339-mk341*	2.4	3.63	0.31	18.88	OD	3.964	0.003**
Fresh root yield	*c1.loc54*	1	mk001-mk035	54	3.09	− 3.85	− 1.06	A	2.829	0.02*
	*c4.loc57.2*	4	mk175-mk181	57.2	4.87	− 4.32	− 4.64	D	6.795	6.7e−05***
	*c7.loc13*	7	mk263-mk264	13.0	4.56	− 4.31	23.48	A	4.619	0.0013**
Harvest index	*c2.loc84*	2	mk083-mk095	84.0	3.38	− 0.01	0.079	A	3.909	0.004**
	*c3.loc84*	3	mk124-mk145	84.0	3.84	0.01	− 0.074	A	3.805	0.0046**
	*c4.loc57*	4	mk179-mk181	57.0	4.83	− 0.02	− 0.14	OD	5.392	0.0005***
	*c5.loc64*	5	mk204-mk205	64	4.35	0.016	− 0.170	A	3.946	0.0037**
Leaf retention	*c6.loc31*	6	mk243-mk245	31	3.52	− 0.003	0.033	A	6.044	0.0004***
	*c21.loc0*	21	mk515-mk516	0	3.95	2.814	2.806	D	6.838	0.0002***
Root number	*c3.loc7.0*	3	mk124-mk129	7	3.87	0.189	− 6.390	A	7.044	0.00108**
	*c7.loc13*	**7**	mk263-mk264	13.0	3.21	2.638	9.841	OD	5.468	0.00480**
Scarlevel	*c2.loc86*	**2**	mk093-mk096	86.0	3.61	19.99	− 39.42	A	2.77	0.017*
	*C5.loc93.9*	**5**	mk219-mk223	93.9	3.31	20.48	− 37.30	A	3.07	0.016*
	*C6.loc0.0*	6	mk232-mk254	0.0	3.25	198.36	185.89	D	2.30	0.035*
Plant vigour	*c3.loc63*	3	mk124-mk141	63.0	3.78	0.05	− 0.97	A	7.16	0.00018***

*a = Traits, b = individual QTLs, c = chromosome where the markers qtls are located, d = Additive gene effects estimated by QTL cartographer, e = dominant gene effects estimated with QTL cartographer, f = marker significantly associated with trait variation, g = probability of the association between a QTL and marker. When a QTL-marker association is significant at more than one trial the most significant P value is declared and corresponding PVE and phenotypic effects of QTLs are given. Gene action was estimated by (d)/(a), OD = over dominance, D = dominance, A = additive, PD = partial dominance, *P = ≤ 0.05, **P = ≤ 0.001, *** = P ≤ 0.0001.

#### Physiologically implied traits

##### Plant height

A trait which is often linked to good growth, was found in this study and analysis to have resulted in the identification of one QTL (*c18.loc19*), implying that the QTL was located on chromosome 18 at 19 cM. The QTL accounted for 7.298% of the phenotypic variation. The allele influencing this trait (positive effect and significant at *P* ≤ 0.001) was from TMS98/0505. The QTL showed an over dominance gene action.

##### Height at first branching (HFB)

**Figure 4:**
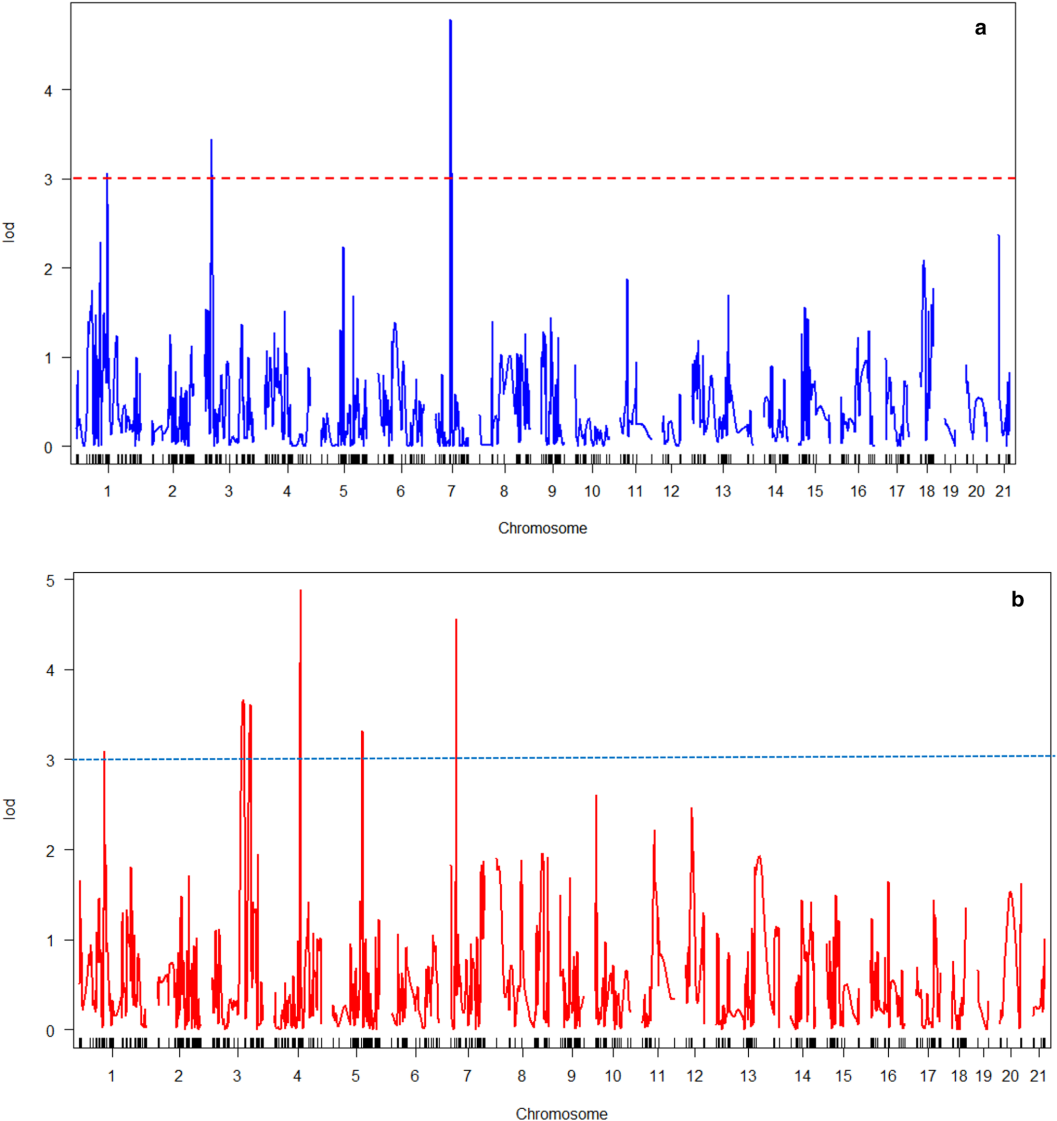
(**a**) QTL peaks for linkage group (LG) 1–21 (height at first branching). (**b**) QTL peaks for linkage groups 1–21 (fresh root yield).

This trait principally defines the architectural outlook of the plant and could condition the amount of foliage associated with plant development. They attained QTL peaks at linkage groups 1–21 with some LoD scores ≥ 3(Fig. 4a). Results indicated three QTLs with additive effects were found on three chromosomes (1, 3 and 7). The phenotypic variation explained by these QTLs ranged from 4.5 to 8%. The alleles significantly influencing HFB originated from the parent TMS98/0505. The gene action for this trait was consistently additive but with a negative effect showing that these QTLs reduced height at first branching. TMS98/0505 is typically a low–medium HFB variety.

##### Branching level (BL)

Analysis identified two QTLs *(C13.loc11.6 and C21.loc 0)* located on LG 13 and 21. The two QTL alleles responsible for increased phenotypic values for this trait in the population were from both parents (i.e. one QTL from either parent). The allele for *C13.loc11.6* originated from TMS98/0581 and was mapped in the interval mk381-mk383 which explained 19.19% of phenotypic variance thus making it a major QTL. The allele in respect of *C21.loc 0* originated from TMS98/0505 similarly explained 20.42% of phenotypic variance observed making it a second major QTL for this trait. The two QTLs detected for branching level expressed different gene actions. QTL *C13.loc11.6* showed over dominance gene action while *C21.loc0.0* showed additive gene action.

##### Leaf retention (LR)

Two QTLs located on chromosome 6 and 21 were significantly associated with LR at highly significant P-values (Table 2). The phenotypic variations explained by the QTLs were similar (6% for *C6.loc31*) and 6.8% for *C21.loc0.0*). The QTL C6.loc31 expressed additive effects while the second QTL (*C21.loc0.0*) showed dominant gene action.

##### Scar level (SL)

This trait revealed several QTLs in the population. Three regions representing different linkage groups were found to be significantly linked to this trait. The LGs include 2, 5, and 6. The LoD scores ranged from 3.25 to 3.61. The PVE was small ranging from 2.3 to 3.07%. Except for QTL *c6.loc0.0* which exhibited dominant gene action, the other two QTLs (*c2.loc.86* and *c5.loc93.9*) were additive in gene action. The three QTL alleles influencing trait expression originated from TMS98/0505. The three QTLs increased scar levels.

##### Plant vigour

One QTL significantly associated with plant vigour was found on LG 3. The phenotypic variation accounted for by the QTL was 7.16. The QTL allele influencing plant vigor was from parent plant TMS98/0505. The QTL detected for plant vigour showed additive gene action.

#### Productivity traits

##### Dry matter content (DMC)

This trait revealed one QTL in the mapping population. The QTL (*c3.loc67*) controlling DMC was located in LG 3 and accounted for 7.67% of phenotypic variation. The QTL exhibited additive gene action and increased DMC. The favourable allele was derived from genotype TMS98/0505.

##### Dry root yield (DRY)

Five different regions of the genome were found significantly associated with this trait. The QTLs were found on LGs 1, 3, 4, 7 and 10. The phenotypic variance explained (PVE) by these QTLs ranged from 3.3 to 5.9%. Three QTLs (*c3.loc68, c4.loc57* and *c7.loc13*) decreased dry root yield and the alleles involved were derived from TMS98/0581. Two other QTLs (*c1.loc54* and *c10.loc2.37*) increased dry root yield with the favorably alleles coming from parent line TMS98/0505. Except for QTL *c7.loc13* which had additive gene action, the other QTLs exhibited either dominant or over dominant gene action (Table 2). Although results indicated dominant gene action for *c3.loc68*, the possibility of additive gene action for this QTL could also not be excluded.

##### Fresh root yield (FRY)

Three significant QTLs were found for this trait and mapped to the same LGs (1, 4 and 7) as with DRY. Some of the QTL peaks had LoD scores > 3 (Fig. 4b). The phenotypic variation explained by the QTLs ranged from 2.83 to 6.795%. The alleles driving yield were from parent genotype TMS98/0505. All the three (loci) QTLs had decreasing effects. As with dry root yield, three gene actions were associated with this trait. Two QTLs had additive effect while one QTL each was found for dominance gene effect (Table 2). The QTL *c1.loc54* tended to show additivity, one could not rule out the possibility of dominance gene effect at this locus. Although c4.loc57.17 tended to show dominance gene effects, additive gene effects could not be excluded in the QTL. Results also show that QTL *c7.loc13* is additive, but dominance cannot be excluded.

##### Harvest index (HI)

Four QTLs significantly associated with harvest index were identified in LGs 2, 3, 4 and 5. The phenotypic variance explained by these QTLs ranged from 3.8 to 5.42%. Parent genotype TMS 98/0505 contributed QTL alleles which increased harvest index at two loci (*c3.loc84* and *c5.loc64*) and with both having additive effect. The other two loci (*c2.loc84* and *c4.loc57*) had alleles (coming from TMS98/0581) which decreased harvest index. QTL *c4.loc57* influenced trait through additive gene action while *c4.loc57* expressed over dominance effects. Thus three of the four QTLs identified for harvest index had additive gene action. Results show that QTL *c2.loc84* was indicative of additive gene action, dominance gene action cannot be excluded. Although QTL *c4.loc57* was identified for over dominant gene action, additive gene action could not be excluded.

##### Root number (RN)

Two QTLs located in LGs 3 (*c3.loc70*) and 7 (*c7.loc13*) were identified for root number. The phenotypic variance explained by *c3.loc70* is 7% while *c7.loc13* has a PVE of 5.5%. The two loci increased root number with the QTL on LG3 having an additive effect while the QTL on LG 7 had overdominance effect. The QTL alleles influencing this trait were from parent genotype TMS98/0505.

### Digenic interactions amongst detected QTL

Five significant digenic interactions were detected with the LOD values and interaction effect of each pair of interactions being significant. The digenic interactions include additive × additive interaction effect, over dominance × additive effect, additive × dominance interactive effect, dominance and over dominance interactive effect and over dominance and over dominance interactive effects (Table 3). The phenotypic variance explained by each digenic interaction ranging from 3.98 to 35.15% with branching level having the biggest effect.

**Table 3:** Digenic interactions amongst detected QTL.

Trait	QTL interactions	PVE%	F value	Gene action
Branch level	13@11.6:21@0.0	35.15	435.3***	OD*A
Height at first branching	1@68.0:3@15.0	5.88	4.375***	A*A
	3@15.0:7@34.6	4.019	2.916*	A*A
Dry root yield	3@68.0:10@2.4	5.23	4.16**	D*OD
	4@57.2:10@2.4	4.23	3.31*	OD*OD
Leaf retention	6@31.0:21@0.0	3.98	2.72*	A*D
Scar level	3@97.5:21@18.0	4.45	3.44**	A*A

*P = ≤ 0.05, **P = ≤ 0.001, *** = P ≤ 0.0001.

## Discussion

While research efforts have been targeted for cassava productivity under optimum conditions of humid and sub-humid ecologies, genetic improvement for cassava production hasn’t been sufficiently targeted for dry ecologies in Africa, where the crop has assumed high importance in recent years. In contrast, several studies on drought tolerance for improved productivity has been done in Latin America. Cassava yield in the Sudan and Sahel ecologies are still low and require genetic improvement to improve productivity, unfortunately the dearth and inaccessibility of molecular tools/facilities are a major constraint for national breeding programs.

This study was facilitated to strengthen breeding capacity in Africa for the development of improved high yielding drought resilient cassava varieties. The non-inbred parents utilized in this study are elite breeding materials used in breeding program of many African countries to enhance their utility in molecular breeding for useful genes. The goal of this paper was to develop/contribute to robust/genetic tools that can be used to drive marker assisted selection (MARS) for complex (quantitative traits) in cassava. The other key aspect of the study was primarily the availability of KASPar based genetic map in cassava which can now be adapted for rapid genotyping platform.

CMD is the most limiting disease of cassava in Nigeria^29^. Both parental lines were selected for CMD resistance to enhance the chance of developing an F_1_ mapping population with good resistance to the disease. This is to minimize any confounding effects of the disease on QTL mapping. Results indicate that the F_1_ progeny did express good CMD resistance (with over 90% being resistant to CMD) making it suitable for QTL mapping.

The F_1_ population showed good segregation with the identification of transgressives for most of the traits as expected since cassava is a highly heterozygous crop. The F_1_ population generally and for most traits showed superior mean performance than the parents indicating an overall better trait expression (useful complementary gene combination) in the progeny. The F_1_ population therefore revealed good candidate genotypes that could potential be injected in breeding pipeline for possible release as varieties in African sub-regions. Principal component analysis accounted for 99% variation for productivity traits and 74% for physiological traits indicating the study explained a sizeable proportion of variation observed in the variables.

Correlation results showed significant association among a number of productivity traits as well as among morphological/physiological traits. Similarly, significant correlations were observed among both sets of traits. The results observed were similar to previous studies indicating that most of these traits are interrelated and co-jointly determining plant response and performance in the target ecology^11,30,31^. Therefore, these traits are important and could be used in direct selection for yield/productivity improvement in African savannas.

The development of about 2000 KASPar SNP markers under the CGIAR Generation Challenge Programme which supported this study facilitated the opportunity to build a new molecular breeding platform for NARs in Africa for cassava genetic improvement through breeding-by-design. These KASPar SNP markers were the first set of SNP markers availed to African NARs for QTL mapping of productivity traits in drought stress ecology. Results shows that SNP markers were highly polymorphic, and abundant in cassava and very efficient for use in genetic dissection of traits in cassava. With further advancement in molecular biology in recent times, nextgen sequencing technologies is now availing several thousands of SNPs for improved genetic studies of cassava^32^.

The constructed SNP genetic map of 21 Linkage groups (LGs) showed good marker distribution and genome coverage. The average marker interval for the map is 3.69 cM with comparable results to the value reported by^33^. The number of linkage group was more than 18 pairs expected for cassava (2n = 36) showing that the map is not fully saturated, however, the aim of having a marker at every 10 cM interval to support and improve QTL studies and breeding application in African NARs was achieved. This map resulted in a lower marker interval than previously identified with SSR markers used by African NARs. The use of two leading elite parent materials highly used in breeding programs to construct this map holds an important opportunity to explore identified QTLs for trait selection and rapid development of superior genotypes.

This study identified 27 QTLs related to crop performance under moderate stress field conditions. QTLs were identified for 11 traits (Table 2). The ultimate focus is about finding all possible QTLs and exploring them based on multiple QTLs approach (using SNPs) in MARS. It isn’t about finding main effect QTLs only (which works for MAS in oligogenic trait), but which in most cases for complex (polygenic) traits do not give substantial forward breeding. The phenotypic variance explained by the QTLs varied from small to moderate and major effects. The phenotypic variance explained for branching levels by two QTLs was large (> 19% each) indicating this trait could easily be manipulated. Branching levels in cassava is a trait that influences the architecture of the crop and may impact assimilate partitioning (sink-source relationship). High branching levels tend to be associated with low branching height (first forking point) and vice versa. It therefore does affect how well cassava can avoid superfluous growth and efficiently utilize assimilates for productivity. This trait is therefore highly beneficial to improving productivity.

Different gene actions (additive, dominance, and over dominance) were found in this study. Almost 40% of the showed dominance or over dominance gene actions indicating both gene actions are critical to cassava performance supporting generally held views that superiority in field performance of cassava is highly linked to its heterozygosity. Intercrosses are highly explored to take advantage of heterozygosity and to minimize inbreeding depression found in cassava. Being a clonally propagated crop, dominance gene effects (heterotic loci) can easily be fixed in cassava^34^. A high number of QTLs with additive gene action were also found, supporting breeding approaches for selfing and recurrent selection scheme to increase the allele frequencies for such loci. In recent times, breeders are increasingly exploring the use of additive genes. These results thus confirm the basis for a molecular breeding approach to effectively combine both additive and dominance gene variance for articulate breeding.

Some QTLs were found to control more than one trait. For example, QTL *c4.loc57.2* for fresh root yield and dry root yield both on chromosome 4. Similarly, *c7.loc13* was involved for both fresh root yield and dry root yield. *C21.loc0.0* was also involved for branching level and leaf retention (Table 4). The localization of QTLs or gene pleiotropic effects have been reported in other QTL mapping studies^11,17,35–38^ and partly account for the underlying significant correlation observed among yield related traits as well morphological and physiological traits as found in this study. Genetic markers co-associated with multiple traits that correlate with each other can effectively be used to select for these associated traits to improve breeding efficiency^39^. Preliminary information (Ewa et al., unpublished) from continued studies suggests some of the identified QTLs (*c3loc84.0*, *c6loc0.0*, and *c7loc13.0*) in this study are stable for productivity based on QTLxseason data set at the same environment under moderate stress. We are continuing with QTLs studies in multi locations and seasons overtime to identify more QTLs and address seasonal variations and location specific and related QTLxE effects. The multiplication ratio of good cassava planting materials does slow processes involving such validation studies.

**Table 4:** Pleiotropic QTLs for physiological and productivity traits in cassava.

Interval	QTL	Linkage group	Traits
mk515-mk516	C21.loc0.0	21	BL, LR
mk263-mk264	C7.loc13	7	RN, FRY, DRY
mk175-mk181	C4.loc57.2	4	DRY, FRY

The quantitative inheritance associated with complex traits (such as productivity) require that both minor and major QTLs would have to be efficiently harnessed to increase productivity in a recurrent selection scheme driven by aid of markers. These tools therefore offer improved opportunities for resource limited national programs in Africa. This study reflects the first set of analysis done to identify QTLs under drought stress conditions in Nigeria, and for Africa. More QTL analysis will be initiated by the authors to support identification of more QTL for additional environments for different stress conditions (including severe stress situations) to capture yet unidentified QTLs and other interactive effects with the aid of decision software tools to support recurrent selection combining several set of QTLs (haplotypes) per trait in a marker assisted recurrent selection as opposed to few in MAS. The KASPar SNPs developed under the GCP are public goods readily available for use by NARs in Africa. Although genotyping by sequencing (GBS) is rapidly evolving, the capacity to utilize the massive data and access to such technologies could still be complicated and would require more expert support to national programs. Molecular breeding future in Africa would be driven by highly informative QTLs which through successive recombination activities will result in the accumulation of desirable alleles for the development of superior genotypes with good genetic gain for improved productivity in drought-prone environments of Africa.

## Supplementary Information

Supplementary Information.
