# Supplementary material for: KASPar SNP genetic map of cassava for QTL discovery of productivity traits in moderate drought stress environment in Africa
Source: Sci Rep. 2021 May 28;11:11268. doi: 10.1038/s41598-021-90131-8 (PMC8163807; doi:10.1038/s41598-021-90131-8)
Supplement: Supplementary file 1 — Supplementary Information. [file 41598_2021_90131_MOESM1_ESM.docx]

KASPar SNP Genetic Map of Cassava for QTL discovery of productivity Traits in moderate Drought Stress Environment in Africa

**Table5 (supplementary data): Descriptive statistics of physiological and productivity traits population and checks**

| **Traits** | **Skew** | **Min** | **Max** | **Mean** | **SD** |
| --- | --- | --- | --- | --- | --- |
| VIG (1-5) | -0.04 | 1.00 | 5.00 | 3.27 | 0.91 |
| RTWT (kg) | 0.88 | 0.10 | 16.00 | 5.18 | 3.33 |
| BIOM (kg) | 0.70 | 0.20 | 14.80 | 4.43 | 2.59 |
| RTNO | 1.53 | 1.00 | 61.00 | 12.36 | 7.41 |
| FRY (t/h) | 1.14 | 0.50 | 80.00 | 23.67 | 14.65 |
| HI | -0.62 | 0.06 | 0.86 | 0.53 | 0.13 |
| DMC (%) | -0.21 | 17.47 | 45.34 | 31.93 | 4.55 |
| DYLD (%) | 1.25 | 0.17 | 26.51 | 7.67 | 4.73 |
| SCARNO | -0.38 | 9.25 | 186.00 | 106.41 | 28.57 |
| NLVS | 1.19 | 0.00 | 176.67 | 47.92 | 33.46 |
| HFB (cm) | 2.16 | 0.00 | 190.40 | 47.27 | 28.21 |
| SCARLEV (cm) | -0.68 | 0.00 | 242.40 | 122.56 | 60.46 |
| LVRET (%) | 1.29 | 0.40 | 63.82 | 20.79 | 10.22 |
| PLTHT (cm) | -0.02 | 42.00 | 255.00 | 154.01 | 36.49 |
| WTLV (cm) | 1.72 | 0.00 | 38.00 | 9.85 | 5.82 |
| BLEV | 2.35 | 0.00 | 79.00 | 3.14 | 5.38 |

**VIG= plant vigour, RTWT= root weight, Biom= plant total biomass, RTNO= root number. FRY= fresh root yield, HI= harvest index, DMC= dry matter content, DYLD= dry root yield, SCARNO= number of scars, NLVS= number of leaves, HFB= height at first branching, LVRET= leaf retention**.

**Table6 (supplementary data): Mean values of traits in parents and progenies**

| **Traits** | **Parents** | **Progeny** |
| --- | --- | --- |
| VIG  BLEV  SCARLEV | 3.38  1.60  **112.6** | 3.46  2.10  87.99 |
| RTWT | 5.20 | 15.05 |
| BIOM | 4.46 | 10.85 |
| RTNO | 12.42 | 27.63 |
| FRY | 23.99 | 33.96 |
| HI | **0.53** | 0.37 |
| DMC | **31.33** | 27.30 |
| DYLD | 7.63 | 8.77 |
| PLTHT | 123.67 | 141.82 |
| SCARNO | 70.83 | 74.95 |
| NLVS | 81.98 | 75.18 |
| HFB | 35.72 | 51.66 |
| LVRET | 20.67 | 26.35 |
